# Deformities of the Globus Pallidus are Associated with Severity of Suicidal Ideation and Impulsivity in Patients with Major Depressive Disorder

**DOI:** 10.1038/s41598-019-43882-4

**Published:** 2019-05-16

**Authors:** Kiwon Kim, Jeong-Hyeon Shin, Woojae Myung, Maurizio Fava, David Mischoulon, George I. Papakostas, Kwan Woo Choi, Eun Jin Na, Sang Won Seo, Joon-Kyung Seong, Hong Jin Jeon

**Affiliations:** 1 Department of Psychiatry, Veteran Health Service Medical Center, Seoul, Republic of Korea; 2 Department of Psychiatry, Depression Center, Samsung Medical Center, Sungkyunkwan University School of Medicine, Seoul, Republic of Korea; 3 School of Biomedical Engineering, College of Health Science, Korea University, Seoul, Republic of Korea; 4 Department of Neuropsychiatry, Seoul National University Bundang Hospital, Seongnam, Republic of Korea; 5 Depression Clinical and Research Program, Massachusetts General Hospital, Harvard Medical School, Boston, MA USA; 6 Department of Neurology, Samsung Medical Center, Sungkyunkwan University School of Medicine, Seoul, Republic of Korea; 7 Department of Health Sciences & Technology, Department of Medical Device Management & Research, and Department of Clinical Research Design & Evaluation, Samsung Advanced Institute for Health Sciences & Technology (SAIHST), Sungkyunkwan University, Seoul, Republic of Korea

**Keywords:** Social behaviour, Human behaviour

## Abstract

Neuroimaging research increasingly suggests there are biological features related to suicidal risk, including brain morphometric features, leading to an elaborate suicide risk assessment. However, few studies have focused on the severity of suicidal ideation and its association with subcortical anatomy in patients with major depressive disorder (MDD). Here, we mainly investigated whether specific structural differences were present in MDD patients with and without suicidal ideation; and supplemented comparison with and without suicidal attempt. We hypothesized that structures associated with suicidal ideation would be derived from a combination of depression and impulsivity. Local atrophy of subcortical structures in 48 patients with MDD (24 with suicidal ideation and 24 without) and 25 age- and sex-matched healthy controls were compared using a surface-based shape analysis method. There was no difference in brain volume between MDD patients with or without suicidal ideations; or MDD patients with or without suicidal attempt. However, the atrophy level in the left pallidum showed a positive correlation with severity of suicidal risk in MDD patients with suicidal ideation. Local atrophy of the left hippocampus, right caudate, and right pallidum had a positive correlation with total impulsivity. These findings possibly suggest that vulnerability to suicidal attempt can be derived from suicidal ideation combined with depression and impulsivity, related to reduced motivational control.

## Introduction

Although suicide prevention has been thoroughly studied, suicide remains a major cause of morbidity and mortality worldwide. Worldwide, suicide resulted in 800,000 deaths in 2018. Every 40 seconds, the suicide death rate increases; it has risen 4% internationally in the past 10 years^1^. Major depressive disorder (MDD) is an important illness associated with suicidal ideation and completed suicide^2–4^. However, it is unclear why many patients with MDD have suicidal ideation.

Suicidal risk has been considered as a continuum^5^ that includes suicidal ideation, plans, attempts, and completion. Growing findings from large cohorts investigating the prevalence of suicidality support the presence of these phenomena across diagnostic entities^6,7^. Previous studies have reported that many risk factors for suicidal behaviors can predict suicidal ideation, but not the transition from ideation to attempt^8,9^. However, considering the associations between suicidal ideation, behaviors, and completed suicide^10,11^, examination of biological correlations of suicidal ideation is important. Recent studies suggest that brain dysfunctions such as impairment in functional connectivity^12,13^, reduced white matter tracts^14^, and decreased gray matter density^15^ in the frontal-subcortical network may impact suicidal ideation in patients with MDD.

Magnetic resonance imaging (MRI), frequently used in studies of structural and functional characteristics, is a noninvasive method to explore the neural correlates of suicidal risk *in vivo*. Only a few structural brain imaging studies have examined the relationship between subcortical structures and suicidal ideation in patients with MDD, and a recent meta-analysis reported small and non-significant reductions in subcortical and intracranial volumes in MDD^16^. Subcortical volumetric correlation with suicidal risk, including a history of suicidal ideation to attempt, has been reported. Findings include reduced volumes of the orbitofrontal cortex, caudate, and pallidum^17^, and increased volume in the right hippocampus^18^ and amygdala^19^ in patients with MDD compared to healthy controls. However, few previous studies have focused on the severity of suicidal ideation and its association with subcortical anatomy in MDD patients.

Therefore, the objective of the present study was to elaborate on the neural basis of suicidal ideation in patients with MDD. We compared participants with or without suicidal ideation, and MDD patients with or without suicidal ideation, to adjust for structural characteristics of MDD. We hypothesized that MDD patients with suicidal ideation would display different regional brain volumes than MDD patients without suicidal ideation. We expected the volumes of these brains regions to be positively correlated with severity of suicidal ideation and impulsivity, which could be validated by suicidal attempt. We also hypothesized that structural regions correlated with suicidal ideation would be independent of those correlated with MDD; such differences may be responsible for the decision-making impairment that accompanied to depression leading to suicidal attempt^20,21^.

## Methods

### Subjects

A total of 48 patients (5 males and 43 females) with MDD were recruited from the outpatient clinic of the Depression Center of Samsung Medical Center, from April 2011 through April 2013. All patients were clinically referred, and none had received psychotropic medications within 2 weeks of the study or fluoxetine within 4 weeks. Inclusion criteria were: 18 years of age or older and experiencing a current unipolar major depressive episode as verified by the *Diagnostic and Statistical Manual of Mental Disorders, Fourth Edition* (DSM-IV) criteria for MDD^22,23^. The diagnosis was based on clinical evaluation by a board certified psychiatrist and the full version of the Mini-International Neuropsychiatric Interview (MINI)^24^. The baseline minimum 17-item Hamilton Scale for Depression (HAM-D)^25^ score required for enrollment was 16. Exclusion criteria were psychotic disorders (*e.g*., schizophrenia or delusional disorder), bipolar affective disorder, neurological illness including significant cognitive impairment or Parkinson’s disease, mental retardation, significant medical conditions, epilepsy, history of alcohol or drug dependence, personality disorders, or brain injury.

Additionally, 25 healthy volunteers with no history of psychiatric disease were recruited by advertisement as a control group. Volunteers with a positive family history of mood disorder were excluded. The study protocol was approved by the Ethics Review Board of Samsung Medical Center, Seoul, Korea. All research was performed in accordance with relevant guidelines. Signed informed consent was obtained from all participants.

### Clinical evaluation

We divided the MDD group into participants with and without suicidal ideation. We also compared MDD participants with and without suicidal attempt. Participants with suicidal attempt included those with a self-injury history within 1 month and those with a previous lifetime suicide attempt. At entry, the intensity of suicidal ideation was assessed with the Beck Scale for Suicide Ideation (SSI)^26^. The SSI is a 19-item self-report measure designed to assess the current attitude, behaviors, and plan to commit suicide over the past week. All items are rated on a 3-point scale of intensity and generate a total score between 0 and 38. The results of the SSI were confirmed by a psychiatrist through a clinical interview. The Korean version of the SSI shows acceptable reliability and validity^27^. Among the 48 patients, 24 were classified as being part of the ‘suicidal ideation group,’ and 24 age/sex-matched patients with MDD who had no suicidal ideation were designated as the ‘no suicidal ideation group.’ Participants were also classified into two groups based on suicidal attempt: 13 had suicidal attempt including self-injury, defined as a self-injury history within 1 month or a previous lifetime suicide attempt; and 35 did not have suicidal attempt. Note that the number of participants in the ‘suicidal attempt’ group is lower than the number in the ‘suicidal ideation’ group because some participants with suicidal ideation had no history of suicide attempt (categorized as ‘non-suicidal attempt’). The severity of suicidal risk was measured by both the MINI-Suicidality Scale^28^ and SSI^29^. The severity of depression and anxiety was measured using the 17-item HAM-D^25^ and the Hamilton Anxiety Rating Scale (HAM-A)^30^. In addition, the Mood Disorder Questionnaire (MDQ)^31^ was used to detect symptoms of bipolarity in depressed patients^32^. The SSI, HAM-D, and HAM-A were administered by a single trained rater at baseline, after 1 month, and after 3 months.

### Image acquisition

Structural images of the brain were acquired from all 48 patients and 25 healthy subjects at the Samsung Medical Center using the same 3.0-T MRI scanner (Philips 3.0 T Achieva) within 1 week after the baseline visit. T1-weighted MRI data were recorded using the following imaging parameters: 1-mm sagittal slice thickness, over contiguous slices with 50% overlap, no gap, repetition time (TR) of 9.9 ms, echo time (TE) of 4.6 ms, flip angle of 8°, and matrix size of 240 × 240 pixels. Images were reconstructed to 480 × 480 over a 240-mm field of view.

### Image processing

To construct surface meshes of subcortical structures, the T1 images were processed to obtain anatomical parcellations using the FreeSurfer software package (version 5.1.0, http://surfer.nmr.mgh.harvard.edu). After parcellation, the labeled images were transformed to the native anatomical space of the input MR data. The subcortical surface meshes were then extracted from the labeled images for each subject by employing a Laplacian-based surface modeling system^33^. Surface-based registration was achieved by adopting a previously developed method to establish the vertex correspondence of subcortical surface meshes across all samples^34^. For given vertices on a subcortical surface mesh, we measured local shape volume (LSV), employing the method proposed by Shapira *et al*.^35–37^. By definition, the LSV measures the amount of local shape volume at each vertex, which can be used to analyze surface-based atrophy of subcortical structures. Each subcortical structure was composed of 2,562 vertices.

### Statistical analysis

Clinical and demographic profiles are presented as categorical variables and continuous variables, as appropriate. Categorical variables included frequencies and proportions. Continuous variables included mean ± standard deviation (SD) or median and interquartile range. Depending on the normality of the distribution, Student t tests, one-way ANOVAs, Wilcoxon rank-sum tests, or Kruskal-Wallis tests were used.

To compare the local shape volumes of subcortical structures among the three groups, we used permutation-based ANCOVA, controlling for the effects of age, sex, level of education^38^, and intracranial volume (ICV)^39^. We re-populated the datasets *N-1* times by random re-assignment (permutation) of all subjects into one of the three groups, where *N* is the number of permutations. We computed *F*-values for the original dataset and *N-1* permuted sets through a simple ANCOVA to form a null distribution of group difference. Then we estimated the significance level of group difference as the fraction of the occurrences whose *F*-values were not less than the *F*-value of the original dataset. We used 5,000 as *N*. We also performed pairwise comparisons for the three groups using permutation-based ANCOVA and the False Discovery Rate (FDR) procedure.

We further performed the correlation test between clinical measurements and the LSV of each subcortical structure. Since the LSV often does not follow a normal distribution, we used Spearman partial correlation to control for the effects of age, sex, level of education, and ICV. For multiple comparison correction, we used cluster-based statistics^40^.

## Results

### Demographic results

Table 1 summarizes the demographic and clinical characteristics of the participants. Forty-eight had moderate to severe depression based on an initial median HAM-D score of 19. Eight of the 13 (33.3%) patients in the MDD group with suicidal ideation had a history of previous suicide attempts. Patients with suicidal ideation had much higher suicidal risk scores as measured by the MINI Suicidality Scale than patients without suicidal ideation, and six patients without suicidal ideation had a suicidality score of 1. The median score of the MINI suicidality scale was 3.5 in all MDD patients. Patients with suicidal ideation had more previous suicide attempts and higher SSI scores than patients without suicidal ideation. There was no significant difference in gender, age, education, number of episodes of MDD, duration of current episode, BIS score, HAM-A score, HAM-D score, and MDQ score between the patient groups. There was also no significant difference in demographic profiles between the two depressive groups and the healthy control group.

**Table 1:** Clinical characteristics.

Characteristics	Total	Suicidal ideation (*n* = 24)	No suicidal ideation (*n* = 24)	Healthy controls (*n* = 25)	*P*
Gender, male (%)*	13 (17.8%)	3 (12.5%)	2 (8.3%)	8 (32.0%)	0.119
Age, years^†^	55 (49.5, 61)	55.5 (46, 62)	55.5 (49.25, 62.25)	55 (51, 59.5)	0.952
Education, years^‡^	11.31 ± 4.32	11.42 ± 4.62	11.25 ± 3.71	11.26 ± 4.73	0.989
Previous attempt history (%)*	8 (11.0%)	8 (33.3%)	0	—	0.004
Number of episodes^§^	2 (1, 2)	2 (1, 2)	1 (1, 2)	—	0.199
Duration of current episode, years^§^	0.6 (0.2, 1.93)	0.6 (0.6, 2.87)	0.65 (0.70, 2.22)	—	0.971
Suicidality score^§^	3.5 (0, 11)	11 (10.37, 16.71)	0 (0.063, 0.437)	—	<0.0001
SSI^§^	11 (2, 17.5)	15.5 (12.8, 20)	3 (3.03, 8.63)	—	<0.0001
**BIS**					
Motor^¶^	15.42 ± 4.52	15.38 ± 5.06	15.46 ± 4.01	—	0.950
Attention-cognitive^¶^	13.58 ± 3.42	14.13 ± 3.93	13.04 ± 2.81	—	0.277
Non-planning^¶^	20.15 ± 5.28	19.96 ± 6.04	20.33 ± 4.51	—	0.809
HAM-D^§^	19 (17, 22)	20 (18.99, 22.8)	18 (17.15, 20.18)	—	0.129
HAM-A^¶^	17.04 ± 4.42	18.13 ± 5.46	15.96 ± 2.76	—	0.090
MDQ^¶^	4.21 ± 3.37	4.33 ± 3.76	4.08 ± 3.01	—	0.800

SSI, Scale for Suicide Ideation; BIS, Barrett impulsiveness scale; HAM-D, Hamilton depression rating score; MDQ, Mood Disorder Questionnaire

*Fisher’s exact test was used.

^†^Kruskal-Wallis test was used; Data are given as median and interquartile range.

^‡^One-way ANOVA was used; Data are given as mean and standard deviation.

^§^Wilcoxon rank-sum test was used; Data are given as median and interquartile range.

^¶^Student’s *t-*statistics was used; Data are given as mean and standard deviation.

### LSV differences in the between-group comparisons

Differences in the LSV between MDD patients with or without suicidal ideation using permutation-based ANCOVA are presented in Table 2. Twelve local regions in the subcortical hemispheres showed differences between the two groups, including the left and right amygdala, caudate, hippocampus, pallidum, putamen, and thalamus. Comparison of the LSV between MDD patients with or without suicidal attempt is presented in Table 3. As shown in Tables 2 and 3, the local regions that showed a difference in LSV with the presence of suicidal ideation partially corresponded to the regions that showed a difference in LSV with the presence of suicidal attempt except both amygdala and pallidum Both amygdala and pallidum showed no difference in LSV with the presence of suicidal attempt. However, these regions showed no significant association with MDD accompanying suicidal ideation after post hoc analysis (covariates of age, sex, ICV, and years of education).

**Table 2:** Comparison of subcortical local shape volumes between groups.

Subcortical structure		2-Group ANCOVA	3-Group ANCOVA & Post-hoc test		
		*MDD* vs. *HC*	*MDD_si* vs. *HC*	*MDD_nsi* vs. *HC*	*MDD_si* vs. *MDD_nsi*
Left	Amygdala	382	360	591	0
	Caudate	710	639	123	0
	Hippocampus	60	462	92	0
	Pallidum	20	109	112	0
	Putamen	2428	2323	2167	0
	Thalamus	610	627	570	0
Right	Amygdala	0	288	0	0
	Caudate	429	367	262	0
	Hippocampus	57	377	96	0
	Pallidum	624	331	303	0
	Putamen	2417	2342	2262	0
	Thalamus	0	0	0	5

*MDD_si*, MDD patients with suicidal ideation; *MDD_nsi*, MDD patients without suicidal ideation; *HC*, Healthy controls.

Each subcortical structure was composed of 2,562 vertices. We showed significant vertex size (FDR-corrected p < 0.05).

**Table 3:** Comparison of subcortical local shape volumes between groups.

Subcortical structure		2-Group ANCOVA	3-Group ANCOVA & Post-hoc test		
		*MDD_s* vs. *Other*	*MDD_s* vs. *HC*	*MDD_ns* vs. *HC*	*MDD_s* vs. *MDD_ns*
Left	Amygdala	0	0	724	0
	Caudate	0	403	200	0
	Hippocampus	0	450	151	0
	Pallidum	0	0	197	0
	Putamen	0	2251	2263	0
	Thalamus	0	570	618	0
Right	Amygdala	0	0	206	0
	Caudate	0	212	403	0
	Hippocampus	0	298	211	0
	Pallidum	0	0	602	0
	Putamen	0	2273	2311	0
	Thalamus	0	0	0	1

*MDD_s*, MDD patients with suicidal attempt (previous suicide attempt or self-injury); *MDD_ns*, MDD patients without suicidal attempt; *HC*, Healthy controls; *Other*, MDD patients without suicidal attempt and HC.

Independent of suicidal risk, an LSV comparison between the MDD patients and healthy participants is presented in Table 2. Compared with the healthy participants, MDD patients showed significantly different right and left putamen volumes. Among these regions, only the bilateral putamen showed significant volume differences between the MDD patients and normal participants.

### Correlations between LSV and clinical measurements

**Figure 1:**
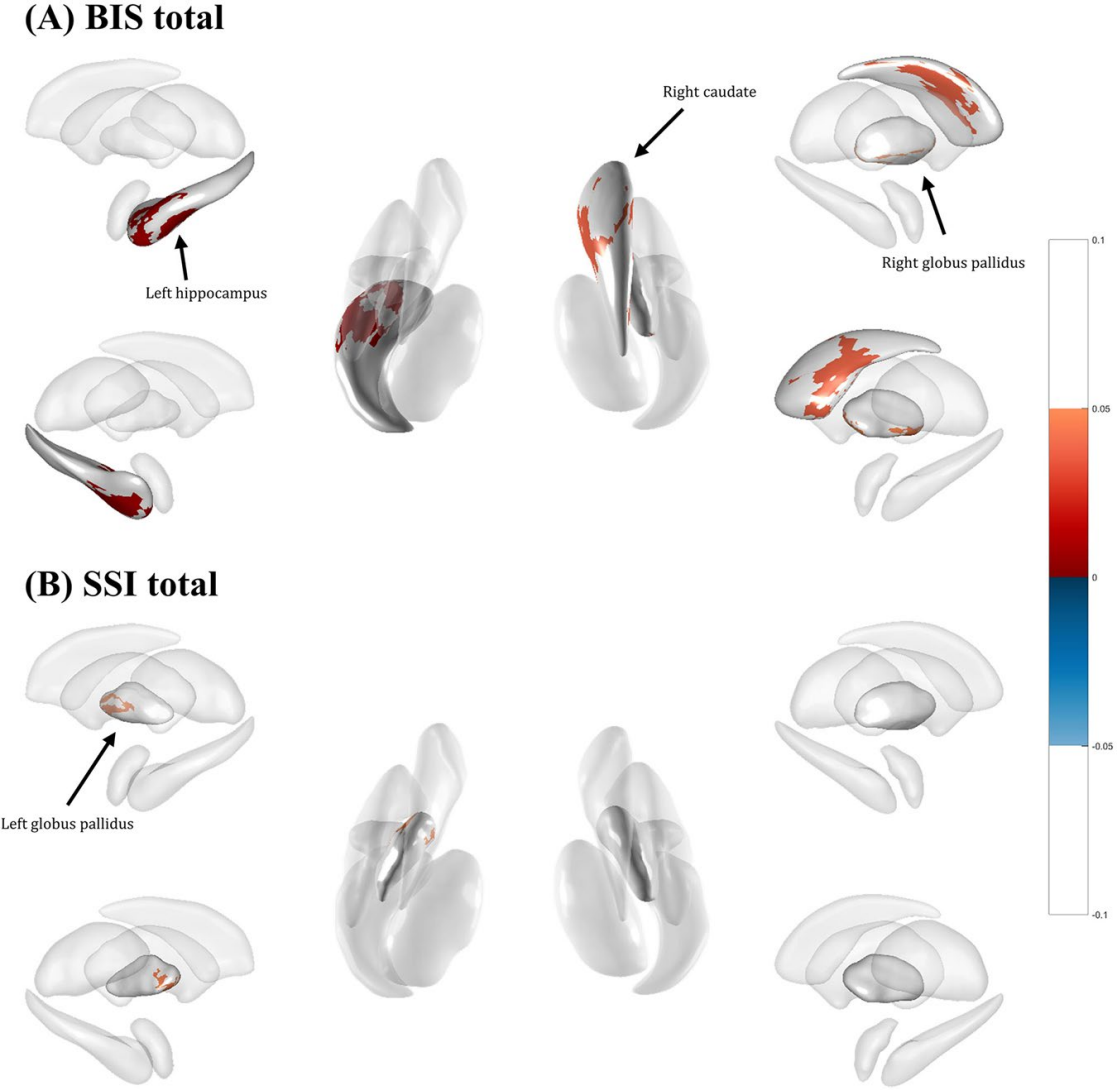
Subcortical structures associated with impulsivity and suicidal ideation. (**A**,**B**) Subcortical structures associated with total impulsivity and structures associated with severity of suicidal ideation are depicted in A and B with negative correlation represented as blue color and positive correlation represented as red color.

Local regions that showed significant correlations with severity of suicidal ideation and impulsivity are presented in Fig. 1 and Table 4. Both thalamus volumes were positively correlated with non-planning impulsivity and lack of future planning and forethought. Both hippocampi were positively correlated with attentional impulsivity, while the volume of the right hippocampus was positively correlated with motor impulsivity, the tendency to act without thinking. The left hippocampus, right caudate, and right pallidum were positively correlated with total impulsivity (Fig. 1A). Interestingly, regions correlated with non-planning impulsivity, motor impulsivity, attentional impulsivity, and total impulsivity were observed with no overlap, except the left hippocampus and right caudate. The left hippocampus volume showed opposite directional correlation with attentional impulsivity and total impulsivity in MDD patients with and without suicidal ideation. The right caudate showed the same directional correlation with total impulsivity in both groups of MDD patients with and without suicidal ideation. The LSV of the left pallidum showed a positive correlation with severity of suicidal risk in MDD patients with suicidal ideation (Fig. 1B). It also showed a negative correlation with severity of suicidal risk in MDD patients without suicidal ideation. However, these regional volumes were not significantly different between MDD patients with or without suicidal ideation after post-hoc analysis.

**Table 4:** Correlations between subcortical local shape volume and clinical measurements.

Clinical measurement	Subcortical structure	*MDD*			*MDD_s*			*MDD_ns*		
		*r*	cluster size	*P*	*r*	cluster size	*p*	*r*	cluster size	*p*
BIS (non-planning impulsivity)	L. Pallidum							−0.3	581	0.0400
	L. Putamen	0.3	414	0.0246				0.3	2409	0.0016
	L. Thalamus				0.3	974	0.0406			
	R. Amygdala							0.3	1549	0.0318
	R. Putamen							0.3	2355	0.0022
	R. Thalamus	0.3	546	0.0108	0.3	1295	0.0144			
BIS (motor impulsivity)	R. Caudate	0.3	313	0.0178						
			258	0.0242						
	R. Hippocampus				0.3	526	0.0408			
BIS (attentional impulsivity)	L. Hippocampus				0.3	875	0.0136	−0.3	770	0.0230
	L. Thalamus							0.3	1573	0.0032
	R. Hippocampus				0.3	522	0.0408			
	R. Putamen							0.3	2108	0.0050
BIS (total impulsivity)	L. Hippocampus				0.3	898	0.0144	−0.3	1196	0.0036
	L. Thalamus							0.3	801	0.0472
	R. Caudate				0.3	816	0.0384	0.3	729	0.0354
	R. Pallidum				0.3	438	0.0494			
	R. Putamen							0.3	1950	0.0152
SSI (severity of suicidality)	L. Pallidum				0.3	427	0.0484	−0.3	529	0.0454
	R. Thalamus							0.3	1108	0.0330
HAM-D (severity of depression)	L. Thalamus							−0.3	970	0.0352
	R. Amygdala				0.3	1698	0.0232	−0.3	1685	0.0252
	R. Pallidum				0.3	651	0.0148			
	R. Putamen				0.3	2162	0.0154	−0.3	1993	0.0128

*MDD_s*, MDD patients with suicidal ideation; *MDD_ns*, MDD patients without suicidal ideation; *r*, correlation coefficient;

*p*, corrected p-value after cluster-based correction; L, Left; R, Right.

In the MDD group, irrespective of the presence of suicidal ideation, the left putamen and right thalamus were positively correlated with non-planning impulsivity. The right caudate was positively correlated with motor impulsivity in the same group. Considering the results from group comparisons between the MDD and normal participants, the correlation between the LSV of the left putamen and non-planning impulsivity was significant. Subcortical structures including the left thalamus, right amygdala, right putamen, and right pallidum showed no significant correlation with depression severity in the MDD group. However, the right amygdala and right putamen were positively correlated with depression severity in MDD patients with suicidal ideation and inversely correlated with depression severity in MDD patients without suicidal ideation. The left thalamus showed a significant negative correlation with depression severity in MDD patients without suicidal ideation.

## Discussion

Our study showed distinct LSV differences between brain regions correlated with the presence of MDD and of suicidal ideation. There was no significant difference in LSV between MDD patients with or without suicidal ideation. Likewise, there was no significant LSV difference between MDD patients with or without suicidal attempt, defined as a self-injury history within 1 month or a previous lifetime suicide attempt. Although a significant group difference was not observed in total subcortical volume, subcortical volumes of specific regions, including the left pallidum, were correlated with suicidal risk severity in MDD patients with suicidal ideation. This directional correlation was positive in the group of participants with suicidal ideation and negative in the group without suicidal ideation. Left hippocampal volume also showed a positive correlation with attentional impulsivity in the group of participants with suicidal ideation and a negative correlation in participants without suicidal ideation.

Our findings agree with a recent meta-analysis that reported no significant differences for any regional brain volume measures between MDD patients with or without suicidal symptoms^16^. The negative correlation between LSV and suicidal ideation may be explained by different components of suicidal risk, including impulsivity, hopelessness, and dysfunction in cognitive flexibility, originating from different brain structures. The small sample size of the current study could also have affected the results. Nonetheless, the finding of differences in the shape of the left pallidum in relation to suicidal ideation may have clinical relevance, if we consider suicidal risk as a continuum. Regions that differed between groups with and without suicidal ideation mostly corresponded to the same regions that differed between groups with and without suicidal attempt except both amygdala and pallidus. This finding supports the validity of previous work relating suicidal ideation to suicidal attempts with negative finding on participants with suicidal attempt especially in amygdala and pallidus^41,42^. Numerous studies have reported the complexity of suicidal risk^43^, in which suicidal ideation and behaviors are transdiagnostic phenomena that can present without distinct diagnosable mental disorders^44,45^. The globus pallidus has been reported to contribute to abnormal temperament, supported by the structural links between parts of the globus pallidus and the reward circuits of the ventral striatum^46^. Among heterogenous suicidal subtypes that reflect various patterns of suicidal thinking and stress responsivity^47,48^, there are consistent findings supporting the importance of the globus pallidus. Recent findings from an animal study^49^ demonstrated the crucial role of the pallidum in top-down control of the cortico-striato-thalamo-cortical circuit to influence perception, attention, and emotion at downstream cortical levels. Thus, even there is limitation to interpret this finding connected to features related to suicidal attempt due to its low sample size, function of top-down control in pallidum would have been correlated to suicidal ideation which lost its correlation after one’s behavior realization through suicidal attempt. However, this study mainly focused on comparison between MDD with suicidal ideation and without, whether than MDD with suicidal attempt and without, which could have lack of evidence to support actual progress from ideation to attempt. There are some of researches reporting volume change of pallidus in suicidal attempt. Increased delay discounting was found to be related to suicide attempts that were poorly planned, of low-lethality, and repetitive. Increased delay discounting was also comorbid with substance use disorders. This behavioral tendency was explained by altered integrity of the basal ganglia^50–52^ and decreased pallidum volume in those attempting suicide^17^. A positive correlation in the left lateralized pallidum with suicidal risk is supported by left lateralization of activity related to cognitive processing^53^. An asymmetric model has also been studied in relation to impulsivity^54^. Increased volume of the right caudate was correlated with increased total impulsivity, consistent with previous findings. The caudate is primarily associated with impulsive, stimulation-seeking features and is observed to contribute to poor decision-making^55^. Considering the pathway supporting adaptive cognitive control, projecting from the anterior cingulate cortex to the striatum and caudate, the same directional correlation observed with total impulsivity in MDD patients with and without suicidal ideation can be understood^56^. We also found that hippocampal volume was correlated with attentional impulsivity, and with left laterality in total impulsivity, consistent with recent research^57^. The relationship between left lateralization and cognitive processing also supports the inverse directional correlations of the left hippocampus between MDD patients with and without suicidal ideation in attentional impulsivity and total impulsivity. Considering the subdomains of impulsivity, a positive correlation with both the thalamus and non-planning impulsivity is in line with previous findings showing a correlation between risk taking and activity in subcortical regions^58^. Likewise, the directional correlation between motor impulsivity and reduced right hippocampal volume is in line with a previous report, showing that dysfunction in this region^59,60^ impacts hyperactivity and impulsivity^61^. However, careful interpretation is needed because our study was not designed to assess asymmetry or altered basal ganglia-thalamo-cortical structure in impulsivity.

Impairment in subcortical structures could facilitate depressive symptoms accompanied by dysfunctional cognitive processes such as poor impulse control. Suicidal ideation with increased intensity could be derived from a combination of depression severity and impulsivity. Top-down control from the pallidus and amygdala which showed volume difference with presence of suicidal ideation could be diminished after realization of suicidal attempt, represented with no difference. Altered paralimbic reward signals and impulsivity with carelessness have been suggested to facilitate unplanned suicidal acts through a disruptive process in the cortico-striato-thalamic circuits^50^. As a result, these findings might suggest that suicidal attempt derived from suicidal ideation can be provoked by reduced motivational control over intentional behavioral reactions to salient negative stimuli in depression^62^. However, limited sample size on participants with suicidal ideation but no history of suicidal attempt and on participants with suicidal ideation with suicidal attempt history, we should be careful to infer these theses. Altered activity in the hippocampal-basal ganglia-midbrain circuit during the salience process has been reported to be correlated with severity of abnormal belief ^63^. Problems related to decision-making and ruminative brooding associated with the salience network are also reported to contribute to suicidal ideation^64^. In the current study, both putamens were demonstrated to show volume changes in MDD. This might reflect deficits in the reward circuit in MDD, which is consistent with recent findings^65,66^. A positive correlation between the right caudate and motor impulsivity in MDD patients and a positive correlation between the left putamen and non-planning impulsivity in MDD patients are consistent with previous reports showing that diminished reward sensitivity is associated with volumetric and functional changes in the nucleus accumbens in MDD^67–69^. Our findings of right laterality in the caudate nucleus and left laterality in the putamen are also in line with previous research^66,70^. Our finding that the left putamen showed significant differences in volume between MDD patients and healthy controls is consistent with recent findings showing that this relationship remains even when controlling for participant age or subtype of depression^66,71^. The lack of a significant difference in caudate volume in MDD patients may have occurred because we excluded cognitively impaired participants, as previous studies have shown an association between smaller caudates and cognitive impairment and dementia^72^. Complete discordance in local volume differences between MDD patients and healthy controls, and volume differences between participants with and without suicidal ideation, agree with previous reports^12,14^.

This study had several limitations. The relatively small number of participants limits the generalizability of the findings. Because we used a cross-sectional design, it was not possible to determine the causal relationships among regional volume changes in MDD patients with suicidal ideation. Despite these limitations, the present study identified differences in regional brain volumes with clinical correlation between participants with or without suicidal ideation in MDD. In addition, corresponding brain regions except amygdala and pallidus differed between MDD patients with or without suicidal ideation also differed between MDD patients with or without suicidal attempt. A strength of this study was the determination of the impact of depression on structural volume between MDD patients with or without suicidal ideation. Although significant group differences were not found in simple group comparisons, a positive correlation between severity of suicidal ideation and impulsivity in structures related to decision-making and reward processes provides evidence for neurobiological markers of suicidal risk in MDD. Thus, consistent with a previous report, the combination of depression with impulsivity related to decision making and the reward process could facilitate suicidal ideation and lead to suicidal attempt^73^. Further study is necessary to elaborate on these findings and to investigate suicide related behavior as a continuum in a transdiagnostic approach. Identification of MDD subjects at particular risk for suicidal ideation and behavior may guide clinicians to use adjunctive psychotherapeutic strategies such as acceptance and commitment therapy, aimed specifically at reducing suicide risk^74^.
